# TSS-EMOTE, a refined protocol for a more complete and less biased global mapping of transcription start sites in bacterial pathogens

**DOI:** 10.1186/s12864-016-3211-3

**Published:** 2016-11-02

**Authors:** Julien Prados, Patrick Linder, Peter Redder

**Affiliations:** 1 Department of Microbiology and Molecular Medicine, Medical Faculty, University of Geneva, Rue Michel-Servet 1, 1211 Genève 4, Switzerland; 2 Laboratoire de Microbiologie et de Génétique Moléculaires, Centre de Biologie Intégrative, Université de Toulouse III, 118 Rue de Narbonne, 31062 Toulouse, France

**Keywords:** TSS-EMOTE, Transcriptome-wide, Transcription initiation, Transcription start site, Sigma factor binding, Promotor usage, *Staphylococcus aureus*, *Staphylococcus epidermidis*, *Acinetobacter baumannii*, *Enterobacter aerogenes*

## Abstract

**Background:**

Bacteria rely on efficient gene regulatory mechanisms to switch between genetic programs when they are facing new environments. Although this regulation can occur at many different levels, one of the key steps is the initiation of transcription. Identification of the first nucleotide transcribed by the RNA polymerase is therefore essential to understand the underlying regulatory processes, since this provides insight on promoter strength and binding sites for transcriptional regulators, and additionally reveals the exact 5’ untranslated region of the transcripts, which often contains elements that regulate translation.

**Results:**

Here we present data from a novel TSS-EMOTE assay (Transcription Start Specific Exact Mapping Of Transcriptome Ends) to precisely map the transcription initiation sites of four entire transcriptomes. TSS-EMOTE is a variation of the dRNA-seq method, which has been combined with the EMOTE protocol, in order to increase detection of longer transcripts and limit biases introduced by PCR amplification of the Illumina sequencing library. Using TSS-EMOTE, 2018 promoters were detected in the opportunistic pathogen *Staphylococcus aureus*, and detailed consensus sequences could be obtained for the RNA polymerase recognition elements (e.g. sigma factor binding sites). The data also revealed a 94 nt median length of the 5’ untranslated region in *S. aureus*, giving important insights for future work on translational regulation. Additionally, the transcriptomes of three other opportunistic pathogens, *Staphylococcus epidermidis*, *Acinetobacter baumannii* and *Enterobacter aerogenes*, were examined, and the identified promoter locations were then used to generate a map of the operon structure for each of the four organisms.

**Conclusions:**

Mapping transcription start sites, and subsequent correlation with the genomic sequence, provides a multitude of important information about the regulation of gene expression, both at the transcriptional and translational level, by defining 5’ untranslated regions and sigma-factor binding sites. We have here mapped transcription start sites in four important pathogens using TSS-EMOTE, a method that works with both long and 3’-phosphorylated RNA molecules, and which incorporates Unique Molecular Identifiers (UMIs) to allow unbiased quantification.

**Electronic supplementary material:**

The online version of this article (doi:10.1186/s12864-016-3211-3) contains supplementary material, which is available to authorized users.

## Background

Transcription in bacteria is initiated by the RNA polymerase holoenzyme, which recognises specific sequence elements on the DNA. This defines the Transcription Start Site (TSS) and the direction of transcription. The exact sequence of the recognised elements is determined by which one of several exchangeable sigma-factors is part of the particular RNA polymerase complex. For example, the most common “house-keeping” sigma-factor, named σ^70^ in *Escherichia coli* and σ^A^ in *Staphylococcus aureus*, recognises two elements centred approximately 10 and 35 bp upstream of the TSS (see reference [1] for a recent review). Some bacteria have many sigma factors, while only four are known in *S. aureus*: σ^A^, σ^B^, σ^H^ and σ^S^, where the latter two are rarely (if ever) used under laboratory growth conditions [2, 3], whereas σ^B^ appears to be involved in stress response and virulence regulation [4, 5]. The RNA polymerase holoenzyme melts the double stranded DNA from 11 nt upstream (position -11) to 3 bases downstream (+3) of the TSS (+1), and the single-stranded DNA can then be used as template for the addition of tri-phosphorylated ribonucleotides. This initiation starts mainly at a specific position, but sometimes “wobbles” one or two bases up- or downstream [6–8]. The first ribonucleotide retains the tri-phosphorylation, whereas the energy from the αP-βP bond in subsequent ribonucleotides is used to elongate the RNA chain.

The DNA sequences around TSSs have long been recognised as crucial for gene regulation in bacteria [1, 9]. Pinpointing the TSS of an RNA permits the identification of potential binding sites for transcriptional regulators, which often bind to inverted or direct DNA repeats to block the RNA polymerase, and moreover, the sigma-factor that specifies the promoter region can frequently be identified via the sequences of the factor-specific recognition motif. These regulatory signals are highly informative for understanding how the expression levels of an mRNA are regulated, and what factors may lead to increasing or decreasing transcription.

Many bacteria of our environment - or even the normal human microbial flora - can transition from natural co-habitants to invading pathogens when the opportunity presents itself. Such a shift implies a change in expression profile, and a large number of studies over the years have focussed on understanding factors contributing to the transcriptional regulation needed to initiate a novel life-style. TSSs of virulence factors have therefore been painstakingly mapped, one by one (see Additional file 1: Table S1 for examples), in order to identify where transcriptional regulators might bind. Additionally, since co-transcription often results in co-regulation, several efforts have attempted to chart operon-structures on a global scale [10–12]. In the last two decades it has furthermore been recognised that 5' untranslated regions (5’UTRs) frequently form secondary structures that can inhibit or promote translation of the downstream open reading frames (ORF). Moreover, they can form simple hairpin structures that block 5' exoribonuclease digestion of the RNA, or consist of elaborate riboswitches that sense the level of a metabolite and either terminate transcription or sequester the ribosome binding site (RBS) [13].

In order to locate the TSS (or TSSs) of an RNA, it was until recently necessary to examine each transcript individually, using either S1 protection, primer extension or a 5' RACE method ([14] and references therein). One of the latter techniques consists of converting the 5' tri-phosphate of the RNA to a mono-phosphate, using Tobacco Acid Pyrophosphatase, whereupon a synthetic RNA oligo can be ligated to the newly generated mono-phosphorylated 5'-end. Reverse transcription PCR can then be performed using a primer specific for the transcript of interest and a primer specific for the synthetic oligo. The resulting PCR product is then cloned and sequenced, and the exact 5'-end of the original RNA molecule can be identified as the first nucleotide after the sequence of the synthetic RNA oligo. With the advent of high-throughput sequencing, differential RNA-seq (dRNA-seq) was developed to simultaneously map all TSSs in the transcriptome. Briefly, the total RNA is split into two pools, one of which is treated with Terminator Exonuclease, which exclusively digests RNA with a mono-phosphorylated 5'-end, and both pools are then treated with Tobacco Acid Pyrophosphatase and are ligated to a synthetic RNA oligo. Libraries are prepared separately for both RNA pools, and are sequenced with a high-throughput RNA sequencing protocol (454 or Illumina) [14]. The exonucleolytic treatment enriches one pool for tri-phosphorylated RNA, i.e. primary transcripts, which are immune to Terminator Exonuclease, and the relative difference in sequencing coverage between the two pools permits specifically designed software to identify the nucleotide that constitutes the tri-phosphorylated 5’-end of the transcript.

Here we present TSS-EMOTE (Transcription Start Specific Exact Mapping Of Transcriptome Ends), a variation of the dRNA-seq method, which equally identifies TSSs on a global scale, but in a manner that is independent of the length and 3’-end phosphorylation status of the RNA, and which incorporates a molecular identification sequence to remove amplification bias inherent in PCR-reactions. We use TSS-EMOTE to identify TSSs of *Staphylococcus aureus*, where the validity of the method is verified, and in three additional opportunistic bacterial pathogens: *Enterobacter aerogenes, Acinetobacter baumannii* and *Staphylococcus epidermidis*.

## Results and discussion

### Mapping of TSSs

**Fig. 1:**
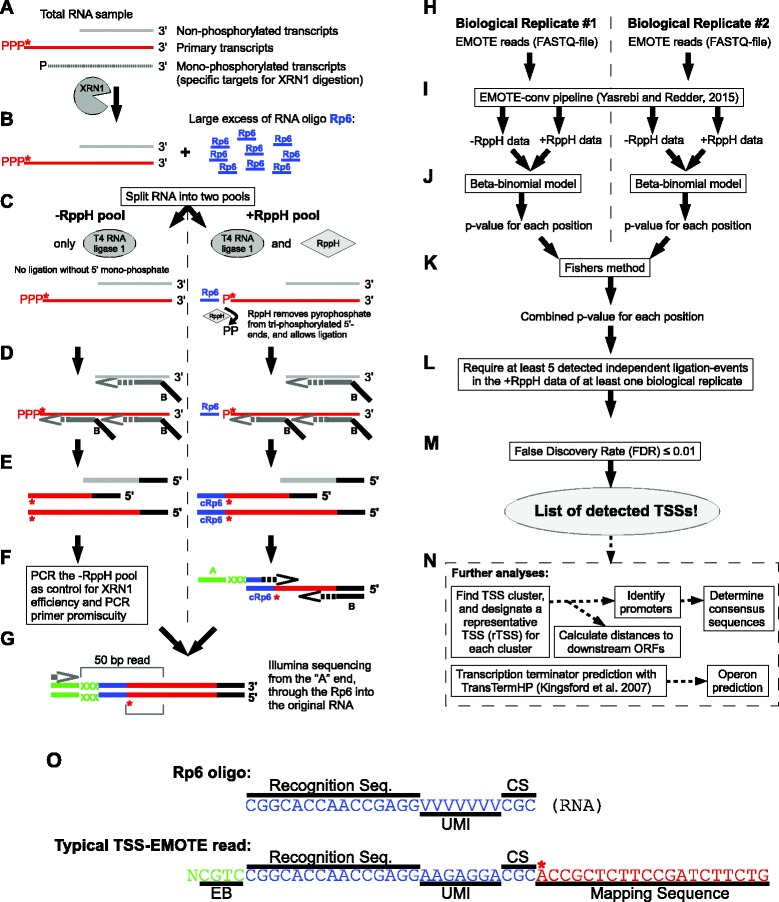
TSS-EMOTE flowchart. The TSS-EMOTE assay consists of a wet-lab library preparation (panels **a** to **g**) and *in silico* analyses (panel H to N). An asterisk continually marks the original 5’-base of tri-phosphorylated RNA (thin red line). **a** Total RNA is purified, and digested with XRN1 5’-exonuclease, which removes the vast majority of 5’ mono-phosphorylated RNA from the sample (including 16S and 23S rRNA). **b** and **c** The XRN1 treated RNA is mixed with large excess of a synthetic RNA oligo (Rp6, shown in blue), and split into two pools. Both pools receive T4 RNA ligase, but only the “+RppH” pool is co-treated with RppH, an enzyme that converts 5’ tri-phosphorylated ends to mono-phosphorylated ends, thus allowing the ligase to use them as substrates. **d** and **e** After the ligation reaction, a semi-random primer is used to reverse-transcribe the RNA and simultaneously add a 2.0 Illumina adapter (black “B”). This results in cDNA with a 2.0 Illumina adaptor (for reverse reads in paired-end sequencing) at the 5’-end and if the template RNA was ligated to an Rp6 oligo, then the cDNA will also have a complementary sequence to Rp6 at the 3’-end (cRp6). **f** PCR is used to specifically amplify cDNAs that carry the 2.0 Illumina adaptor and cRp6 sequences. This step moreover adds a 1.0 Illumina adaptor (for forward reads in paired-end sequencing) and a sample-specific 4-base EMOTE barcode (blue line and “XXX”, respectively) to index the molecules (different barcodes for the -RppH and + RppH pools). The barcode of the -RppH pool will designate molecules where the XRN1 treatments has been incomplete, and this information is incorporated into the *in silico* analysis (see below). **g** The barcoded DNA from various samples (and pools) can be mixed, and loaded directly into an Illumina HiSeq machine. Millions of 50 nt sequences are obtained, each of which will span the EMOTE barcode, both known and random sections of the Rp6 oligo (see Methods), and it will reveal the first 20 nt of the native 5’-end of the ligated RNA molecule. These 20 nt are sufficient to map the vast majority of 5’-ends to a unique position on the small genomes of the bacteria in this study. However, longer Illumina reads (and thus longer mapping sequences) can be used if the TSSs are in repeated regions or if large-genome organisms, such as humans, are being examined. **h** The *in silico* pipeline input consists of stranded RNA-seq reads for one or multiple biological replicates in FASTQ format. Each replicate includes a FASTQ for the -RppH pool and another for the + RppH pool. **i** The FASTQ files go through EMOTE-conv software [51] that parses the reads, aligns them to the genome, and perform the quantification. Thus, for each genomic position we obtain the number of reads whose first nucleotide align at this genomic position, and on which strand it maps. The counts are further corrected for PCR biases by looking at the unique molecular identifiers (UMIs) sequences available in the unaligned part of the EMOTE read. **j** Quantification counts obtained for + RppH and -RppH pools are compared through a beta-binomial model that tests whether the identified 5’ ends in the + RppH pool is significantly enriched over the identified 5’ ends in the -RppH pool at a given position. The process results in a p-value that reflects our confidence in the genomic position to be enriched in the + RppH pool of the biological replicate. **k** The p-values of all the biological replicates are combined into a single p-value with Fisher’s method. **l** and **m** To correct the p-values for multiple testing across all genomic positions, the false discovery rate (FDR) is evaluated and only those with a FDR ≤ 0.01 are considered to be TSSs. Note also that for the FDR is only calculated for genomic positions with at least 5 detected ligation-events in at least one of the + RppH pools (UMI ≥ 5). **n** The TSSs then enter an annotation process that retrieve their surrounding sequence and downstream ORFs. TSSs separated by less than 5 bp are clustered together. Finally, to draw a global picture of operon structures, an independent detection of transcription terminators is operated with the software TransTermHP [39]. **o** Sequence of the RNA oligo Rp6 and a typical Illumina sequencing read from a TSS-EMOTE experiment. The Recognition Sequence serves as priming site for the PCR in panel F. UMI: The randomly incorporated nucleotides in the Rp6 oligo that serves to whether Illumina reads with identical Mapping Sequences originate from separate ligation events. CS: Control Sequence. EB: EMOTE barcode to index the Illumina reads. An asterisk indicates the 5’ nucleotide of the original RNA molecule

To establish and validate our protocol for experimental determination of TSSs, we started out using the community acquired *Staphylococcus aureus* strain MW2 [15], grown in RPMI medium (an artificial serum supplement with a defined composition) at 37 °C. RNA was isolated from exponentially growing cultures (OD_600_ of 0.4) whereupon the Transcription Start Specific - Exact Mapping Of Transcriptome Ends (TSS-EMOTE) protocol (see Fig. 1 and Methods) was used to experimentally determine the exact 5'-ends of the RNA and which of these 5'-ends were tri-phosphorylated. Briefly, the RNA is digested with the 5’-3’ exoribonuclease XRN1, which specifically degrades RNA with mono-phosphorylated 5'-ends but leaves tri-phosphorylated and non-phosphorylated RNA intact (Fig. 1a). The RNA is mixed with a synthetic RNA oligo (Rp6) and then split into two pools: Pool “+RppH”, where both T4 RNA Ligase 1 and the RNA 5’ pyrophosphohydrolase RppH enzymes are added, and pool “-RppH” where RppH is left out (Fig. 1b and c). RppH is able to remove the gamma and beta phosphates from RNA with a tri-phosphorylated 5'-end or m^7^GDP from capped eukaryal RNA [16, 17], converting the RNA to a mono-phosphorylated species allowing the 5’end to be used further. Reverse transcription is then performed on both pools, using a semi-random primer (the DROAA oligo, which was synthesised with a mix of random and defined nucleotides at the hybridising 3’-end) that initiates at close-to-random positions along the RNA, and adds a “Reverse” Illumina sequencing adaptor to the 5’-end of the cDNA (Fig. 1d and e). Then a PCR reaction with primers that are specific for the Rp6 oligo and the “Reverse” Illumina adaptor, is used to amplify the cDNA that originate from ligation products and add a “Forward” Illumina adaptor sequence in extension to the Rp6-sequence (Fig. 1f). The PCR reaction products are then size-selected on a gel (300-1000 bp, Additional file 2: Figure S1). At this point in the protocol, the DNA contains Illumina adaptors at both ends, barcodes, as well as the Rp6 oligo sequence, which adds up to 149 bp. The 300 bp lower limit that is size-selected, therefore correspond to a 151 nt theoretical minimum length of the original tri-phosphorylated RNA. The gel-extracted PCR products are then sequenced from the “Forward” direction in an Illumina HiSeq 2500 machine (50 nt read-length), and each valid read will have an Rp6 sequence, followed by the first 20 nt of the original 5'-end of the RNA that was ligated to the Rp6 oligo. These 20 nt can then be mapped to the genome of the organism, thus indicating the exact base that corresponds to the native 5'-end of the RNA (Fig. 1o). As in the previously reported EMOTE protocol [18], the Rp6 oligo was synthesised with a Unique Molecular Identifier (UMI), a stretch of 7 randomly incorporated bases (either A, C or G), which permits the distinction between Illumina reads arising from a single ligation event that has been amplified by the PCR step, and reads arising from multiple ligation events (and thus multiple original RNA molecules with the same 5’-end). This is achieved by comparing all Illumina reads with identical Mapping Sequences (Fig. 1o), and counting the number of non-identical UMIs, since different UMI sequences must arise from separate ligation events (and not from the same Rp6-ligated RNA which has been amplified by PCR). We therefore use the number of these UMIs, i.e. the number of observed independent ligation events, for further calculations.

T4 RNA Ligase 1 can only use 5' mono-phosphorylated RNA as substrate for ligation, and – assuming that the XRN1 digestion was complete – it is therefore only in the “+RppH” pool that ligation can take place. However, certain secondary structures resulting in recessed 5'-ends will protect RNA from XRN1 digestion (e.g. 5S rRNA and tRNAs). The “-RppH” pool therefore serves to determine the background level of ligation, and avoid false positives. For a given position on the genome, a large number of reads from pool “+RppH” that map to the position is thus indicative of a TSS, but only if the same position exhibits a low (or non-existent) number of reads in the corresponding “-RppH” pool. A beta-binomial statistical test was employed in order to provide a consistent comparison between corresponding + RppH and -RppH datasets, and determine whether the number of reads mapping at a given position on the genome in the “+RppH” pool is significantly higher than the number of reads obtained in the “-RppH” pool. Whenever this was the case, with a false discovery rate (FDR) below 0.01, then the position was designated as a TSS.

### Identifying clusters of TSSs and selecting a single representative TSS per promoter

Using the criteria described above, 2821 TSS positions were identified on the genome of *S. aureus* MW2 in RPMI medium at 37 °C (Table 1). Of these, 1497 TSSs were at isolated positions, whereas 1324 TSSs were within 5 nt of another TSS and clearly grouped into 521 clusters that each presumably arose from the same promoter due to “wobble” of the RNA polymerase initiation. The alternative TSS nucleotides are usually immediately adjacent to each other, but can be up to three bases apart [7, 8]. The clustering of TSSs was carried out by grouping all TSS which were less than 6 bp apart (and on the same strand), and in a few very rare cases, this led to long spread-out clusters (described below), when a TSS would be detected in between two other TSS which were more than 5 bp apart (potentially up to 10 bp), and in these cases it is of course highly improbable that all TSSs in a cluster originate from the same promoter.

**Table 1:** Overview of detected TSSs

	*S. aureus*	*A. baumannii*	*S. epidermidis*	*E. aerogenes*
Growth medium (all at 37 °C)	RPMI	LB	MH	LB
GC content	33 %	39 %	32 %	55 %
Genome size (bp)	2820462	4001457	2564615	5280350
Number of annotated ORFs	2818	3903	2558	5022
Number of detected TSSs	2821	1540	2207	763
Number of TSS clusters	2018	1130	1713	576
Median rTSS-ORF distance (bp)	94	81	98	133

**Fig. 2:**
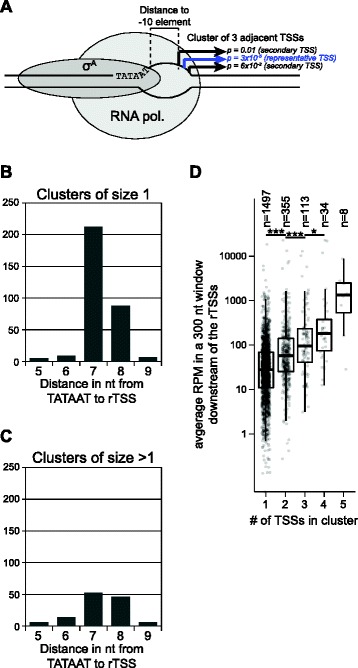
Distances of perfect TATAAT -10 elements to TSS, and the relation between cluster-size and level of gene expression. **a** Cartoon of the transcription initiation complex bound to a promoter, with three potential TSSs. The rTSS (with the lowest p-value) is indicated in blue. **b** Distances from the σ^A^ -10 elements to TSS clusters with a single detected TSS. In order to avoid ambiguity, only TSS clusters that have the perfect σ^A^ TATAAT -10 element consensus sequence are included. **c** Distances from the σ^A^ -10 elements to the rTSS in TSS clusters with more than one detected TSSs. As in panel B, only perfect TATAAT elements are included. **d** Higher expression levels increase the chances of detecting alternative TSSs for a promoter. Expression level from each TSS cluster (a single TSS in the cluster corresponds to an isolated TSS) was estimated by taking the average number of reads in the RNAseq data that map inside a 300 nt window immediately downstream of each rTSS. RPM indicates Reads Per Million. Shapiro-Wilk test was carried out to confirm normal distribution (*p* < 0.05), and Students T-test was used to determine that expression was higher from TSS clusters with wobble (*** and * indicate *p* < 0.001 and *p* < 0.05, respectively). The number of clusters with 5 TSSs was too low to perform a meaningful statistical analysis

In order to proceed with the analyses of the promoters, the 5’-UTRs as well as expression levels, it was necessary to obtain a single representative TSS (rTSS) per promoter, to avoid the same “wobbly” promoter counting multiple times in the statistics. To begin with, the 1497 isolated TSSs (i.e. the only TSS within 5 nt) were automatically designated as rTSSs, and for TSSs detected within the 521 clusters, each TSS position was compared to its neighbours and the TSS with the lowest p-value in each cluster was chosen as the rTSS position (Fig. 2a). To ensure that this method of choosing the rTSSs was justified, all TSSs where a perfect TATAAT -10 element consensus sequence could be identified were extracted. Within this data-set of 624 TSSs, the distances between the rTSSs and their corresponding -10 elements were plotted, and compared to the equivalent distances for isolated TSSs. The most frequent distance from the -10 element was in both cases 7 nt (Fig. 2b and c), which also is the distance that was recently shown for *E. coli* [7], and we therefore conclude that the p-value is a good indicator for selecting a representative TSS for a given promoter.

From the 521 identified TSS clusters, 510 clusters had 2 to 5 TSSs, whereas the remaining 11 clusters showed more than five TSSs (discussed below). To examine whether the number of TSSs in a cluster was correlated to expression level of the RNA, total stranded RNA sequencing (RNAseq) was performed on the same RNA samples used for the TSS-EMOTE assay. The 2007 clusters with five or less detected TSSs, were grouped according to the number of TSSs in the clusters, and the RNAseq read-count that mapped within a window of 300 nt downstream of each rTSS was plotted. As can be seen in Fig. 2d, the level of RNA did indeed increase significantly with the number of TSSs in the clusters, which indicates that the majority of RNAs are transcribed from “wobbly” promoters, but that the minor TSSs often are below detection level for transcripts with lower abundance.

### Evaluation of the TSS mapping

The danger of using global methods (such as TSS-EMOTE) is that it is difficult to evaluate the success- or failure-rate, when the output becomes so large that it is unfeasible to verify everything by hand. In order to evaluate the correctness of the detected TSSs, three main strategies were chosen: i) A search for features that are known to be biologically important for TSSs, ii) comparison to TSSs identified previously in other studies, and iii) a global verification that the TSSs are located in a correct genomic region to be the 5’-ends of RNA molecules.

**Fig. 3:**
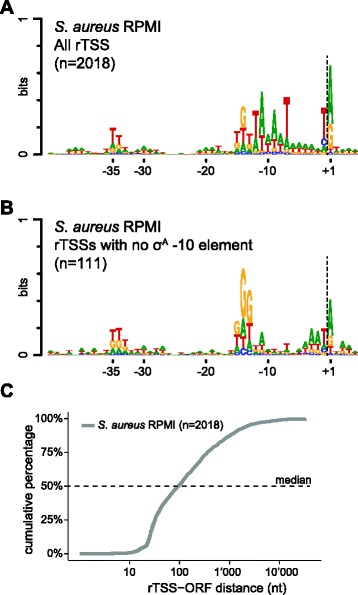
Genomic context of the rTSSs. **a** The genomic sequence surrounding each of the 2018 detected rTSSs (from position -45 to +5, relative to the rTSS) were used to generate a logo-plot (as described in Materials and Methods), without any attempt at alignment of motifs. The -10 element is clearly visible, even without any attempt to align the promoter sequences to optimize the similarity, however, the -35 element is almost indistinguishable, both because it is less conserved than the -10 element, but also because the distance to the TSS is highly variable, causing the signal to dissipate. **b** Logo-plot of the rTSSs where no σ^A^ -10 element could be identified, as difined by more than 2 mismatches to the TATAAT consensus sequence. The logo-plot was generated as described for panel **a. c** Plot showing cumulative percentage of the distance to the nearest (annotated) downstream ORF for each rTSS

The major factor in choosing a specific position on the genome to initiate transcription is the binding of a sigma-factor to the DNA, with each sigma-factor exhibiting its own sequence preference. It was possible to locate a σ^A^ consensus sequence (TATAAT) five to nine nts upstream of 94 % of the rTSSs (1907/2018). 22 % of the rTSSs (450/2018) were perfect TATAAT, with 47 % (953/2018) and 25 % (504/2018) of the rTSSs exhibiting a single and two mismatches, respectively (Additional file 3: Table S2). Moreover, a Logo-plot with all the sequences surrounding detected rTSSs revealed a clear -10 element as well as a preference for T at the -1 position and a purine as the first nucleotide of the RNA (Fig. 3a), a feature which has been recognised for a long time [7, 19].

The remaining 6 % of detected promoters generally corresponded well to the *S. aureus* σ^B^ binding site, and a logo-plot combining all of them exhibited a similar consensus to the *B. subtilis* consensus sequence “GttTww 12–15 gGgwAw” [20] (Fig. 3b) as well as to the small subset of σ^B^-dependent *S. aureus* TSSs which were mapped previously [4, 5] (Additional file 1: Table S1). Furthermore, many of the σ^B^-dependent *S. aureus* genes previously identified by microarray [4] were downstream of one of the 6 % rTSSs with no TATAAT -10 element (Additional file 3: Table S2).

In addition to being downstream of a potential sigma-factor binding site, most TSSs are expected, from a functional point of view, to be immediately upstream of, and on the same strand as, annotated open reading frames (ORFs), although many ncRNAs and pervasive anti-sense transcripts are found in *S. aureus* [21, 22]. Comparing the detected rTSS positions to the annotated genome gives a median distance to the nearest downstream annotated start codon (i.e. the 5’UTR) of 94 nt (Fig. 3c; Table 1), with 64 % of the rTSS at less than 200 bp from a downstream annotated ORF, and only 13 % with a distance of more than 1000 bp (Fig. 3c).

**Fig. 4:**
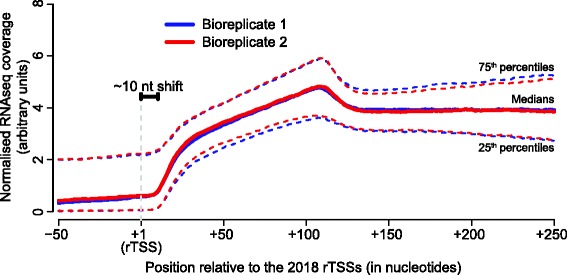
RNAseq coverage is low upstream of the rTSSs and a ~10 nt shift illustrates the loss of 5’-end information in a standard RNAseq protocol. The RNAseq coverage, from Illumina TruSeq stranded RNA sequencing, was determined for each nucleotide in a region from 50 nt upstream (-50) to 250 nt downstream (+250) of each of the 2018 rTSSs identified for *S. aureus*. The median RNAseq coverage from the two biological replicates is shown in red and blue. The dotted lines correspond to the 25^th^ and 75^th^ percentiles, respectively. A grey dotted line indicates the superimposed positions of the 2018 rTSSs, and a black bracket indicates the ~10 nt shift between coverage in the TruSeq protocol and the exact 5’-mapping of the TSS-EMOTE. The traces of RNAseq coverage for each of the 2018 rTSSs is shown in Additional file 2: Figure S1

The ligation event in the EMOTE protocol (Fig. 1c) guarantees that a detected position is a 5’-end of an RNA molecules, but not necessarily a TSS, since it potentially could be a cleavage in the middle of an RNA [18, 23, 24]. Therefore, the next step was to verify that the TSS clusters detected with the TSS-EMOTE protocol do indeed correspond to the original extreme 5’-ends of RNA molecules. To do this, the sequence coverage from the “standard” (i.e. not 5’-end specific) RNAseq data was determined from 50 nt upstream to 250 nt downstream of each of the rTSSs. Averaging these data across all 2018 rTSSs clearly show an increase in RNA levels at the rTSS positions, strongly suggesting that they mark the 5’-ends of RNAs (Fig. 4). Interestingly, we observe a slight shift of about 10 nt, between the rTSSs and the increase in coverage, which underlines that the TruSeq RNA sequencing protocol (used to prepare the RNAseq library for Illumina sequencing) does not preserve the native 5’-end of the RNA. To generate the second stranded cDNA, the TruSeq protocol first cleaves the RNA with RNase H, whereupon DNA Polymerase I uses one or more of the resulting RNA fragments as primer to synthesise the second strand (with dUTP instead of dTTP to ensure strand-information is kept), and finally the 3’ overhang (where the RNA primer used to be) is removed to generate blunt-ended double stranded DNA. The relatively sharp increase in coverage seen around +10 nt in Fig. 4, indicates that this length is either the minimal length of RNA fragment that RNase H can generate or that it is the minimal length of RNA that can be used as primer for the second-strand DNA synthesis step. In either case, irrespective of the ~10 nt shift, the dramatic over-all difference in RNAseq coverage between the regions upstream and downstream of the rTSSs positions (Fig. 4), is consistent with a correct TSS identification in a majority of the 2018 cases.

#### Do the identified TSS correspond to previously identified TSSs?

By searching the literature extensively, we could identify 40 individual and precisely mapped TSSs in *S. aureus*, by either primers extension or 5’-RACE [2, 5, 25–37]. We were able to detect 22 of these 40 TSSs exactly as they were described. Taking into account that primer extension can be slightly imprecise due to inconsistencies in reverse transcriptase reaction, we allowed up to 5 nt discrepancy from the TSS-EMOTE data and were thereby able to increase the number of re-detected TSSs by 7. When TSSs found for alternative growth-conditions (described below) were taken into account, then this number went as high as 32 out of 40. The remaining 8 undetected sites are only expressed under specific growth conditions or in certain strains [2, 25, 28, 33–35], and it is thus not surprising that we were unable to re-detect these (Additional file 1: Table S1).

#### TSS clusters with six or more TSSs

As discussed above, most transcription start sites described so far are clustered at a single position or are a few nucleotides apart. Interestingly, our analysis revealed 11 clusters with six or more TSSs (Additional file 4: Table S3). A single cluster even extended to 33 TSSs, each of which within 5 nt of its neighbour, spanning a region of 48 bp on the genome. The appearance of these ≥6 TSS clusters is rare and we have not identified anything in their genomic contexts that links them (Additional file 4: Table S3), and it is therefore difficult to draw any definite conclusions about what causes them. Possible explanations could be overlapping promoters or promiscuous sigma-factor binding sites, influenced in their binding by local DNA topology [7]. It is interesting to note that among these large clusters, eight of them are highly expressed (i.e. higher than 100 reads in the assay described for Fig. 2d), two drive the expression of phenol soluble modulins [38], and in two clusters it appears that there is an overlap of σ^A^ and σ^B^ recognition sites, which will naturally spread the TSSs over a wider range (Additional file 4: Table S3).

### Alternative growth conditions reveal additional TSSs

Not all transcripts are synthetized at any given growth condition. Therefore, in order to obtain a list of TSSs as complete as possible, *S. aureus* cells were cultured under three alternative growth conditions, in addition to the 37 °C RPMI medium that was used initially: Mueller-Hinton medium (MH) at 37 °C, at 30 °C and on agar plates. The alternative growth conditions permitted the identification of as many as 647 additional TSS clusters (from 1128 additional individual TSS positions; Additional file 3: Table S2), several of which corresponded to sites that had previously been experimentally mapped by other laboratories, but which could not be identified in the TSS-EMOTE data from *S. aureus* grown in RPMI medium (Additional file 1: Table S1). For each of the four growth conditions, it was possible to identify TSS clusters that were unique to a particular data-set, and we presume that these TSSs correspond to transcripts that are only present at detectable levels when the cells experience a particular environment.

### Operon prediction based on TSSs

**Fig. 5:**
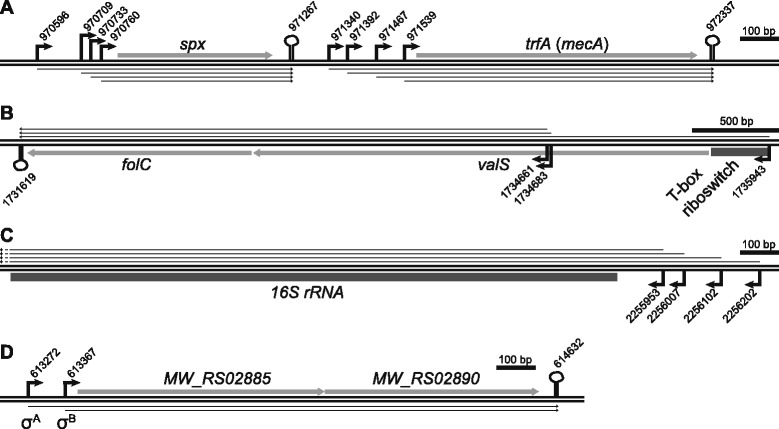
Examples of gene loci and operon predictions. The double-lines indicate the genomic DNA. rTSSs are indicated as bent black arrows, with genomic positions, and RNA as thin black arrows. ORFs are shown as light grey arrows. Transcriptional terminators predicted by TransTermHP [39] are indicated by stylised hairpins. The nucleotide position of each key element is also shown. **a** Layout of the *spx* and *trfA* locus, with multiple TSSs for each ORF (the use of the names *trfA* and *mecA* depend on which *S. aureus* genome annotation is used). **b** The *valS*-*folC* operon. Expression of the valine-tRNA ligase (*valS*) is tightly controlled by a T-box riboswitch in its 5’-UTR (thick dark grey line) that is transcribed from a position 350 nt upstream of the ORF [23]. The transcript continues into the downstream *folC* gene, which seems to be dually controlled by both the T-box riboswitch and by its own promoters at positions 1734661 and 1734683. **c** An example of the beginning of ribosomal RNA operon, where multiple promoters (and TSSs) permit high transcription. Only the first gene of the operon (16S rRNA) is shown as a dark grey line. **d** Example of an operon where separate σ^A^ and σ^B^promoters contribute to expression (indicated underneath their respective bent arrows)

Genes involved in a given pathway or function are often arranged in operons with one common promoter. The TSS data that has been obtained with the TSS-EMOTE assay identifies these promoters (for the examined growth condition), and thus defines the beginning of the operons. In contrast to TSSs, bacterial transcription terminator signals are relatively easily identifiable by bioinformatics analyses of the genome sequence (with the exception of rho-dependent terminators), and a number of tools have been developed for this purpose. In order to generate predicted operon maps, we have here chosen the TransTermHP software [39] to define the termination sites, and combined this with the TSS-EMOTE information and gene annotations from NCBI (http://www.ncbi.nlm.nih.gov/). A few highlights from the generated operon table (Additional file 5: Table S4) are shown in Fig. 5. For example, the separate transcription of the *spx* and *trfA* genes, with multiple TSSs for each gene, that has previously been detected by Northern blotting without detailed mapping (Fig. 5a) [40]. Furthermore, the TSS of the T-box riboswitch which has previously been shown to attenuate the *valS* transcript could be detected [23], with the additional information that the downstream *folC* gene appears to be co-regulated by this riboswitch and its own promoters (Fig. 5b). Additionally, many highly abundant transcripts are expressed from multiple promoters, with up to four of them for rRNA operons (Fig. 5c), and several examples of dual regulation of a single operon by both σ^A^ and σ^B^ promoters (Fig. 5d).

### The application of TSS-EMOTE to other organisms

The TSS-EMOTE protocol was developed and optimised for use with *S. aureus*. However, in order to demonstrate the general utility of TSS-EMOTE, three additional pathogenic bacteria were chosen for TSS mapping. *Staphylococcus epidermidis* was chosen because it is a close relative of *S. aureus*, and shares many of its genomic features. The remaining two pathogens, *Acinetobacter baumannii* and *Enterobacter aerogenes*, are in contrast distantly related γ-proteobacteria that are also important pathogens [41–47].

While many TSS were identified in all examined organisms (Table 1), it is clear that the efficiency of detection varied between the four transcriptomes, and we were notably only able to identify 763 TSSs in *E. aerogenes*. We have three potential explanations (not mutually exclusive) for this: i) in the rich laboratory medium used for growing *E. aerogenes* (LB medium), only a small sub-set of the transcriptome is expressed ii) it is also possible that the high G + C content (55 %) of the *E. aerogenes* genome, leads to secondary structures that prevent the ligation step of the TSS-EMOTE protocol. However, this does seem a somewhat unlikely explanation, since we have successfully performed a related EMOTE protocol on RNA from *Caulobacter crescentus*, which has a G + C content of 67 % [24], and the G + C content is therefore probably not a major obstacle for the ligation step. iii) A more intriguing possibility is that *E. aerogenes* maintains a high ratio of its mRNA in 5’-mono-phosphorylated rather than tri-phosphorylated form, which would prevent all of the various TSS determination methods from being efficient. Since *E. aerogenes* does not encode the tri-phosphorylation-inhibited 5’ to 3’ exoribonucleases RNase J1/J2, which is found in both *S. epidermidis* and *S. aureus* [48], it should be possible to maintain such an mRNA population. This possibility might also account for the relatively low number of detected TSSs in *A. baumannii*, which equally lacks RNase J.

**Fig. 6:**
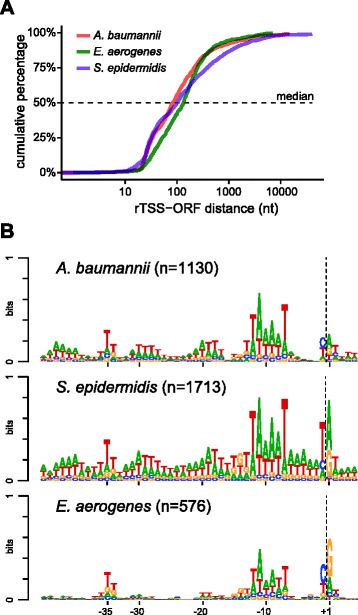
rTSSs in *A. baumannii*, *S. epidermidis* and *E. aerogenes*. **a** Distances from the rTSSs to the start codon of the nearest downstream annotated ORFs. **b** Logo-plots of the promoter regions of the rTSSs reveal clear σ^A^/σ^70^ recognition sequences in all examined organisms (see also Fig. 3a). Dotted lines indicate the beginning of the RNA sequences, and the nucleotide position relative to the rTSS is shown below

Nevertheless, the confidence level for the detected TSS remain high for all organisms, not only due to the calculated p-values, but also due to the fact that each TSS corresponds to at least five independently observed ligation events, and because the TSSs are distributed correctly with respect to the annotated ORFs (Table 1, Fig. 6a). Moreover, we could establish that the TSS-EMOTE assay is highly reproducible (in terms of quantification), by comparing the data of biological replicates for all our strains and growth conditions. Specifically, we compared the number of UMIs in the + RppH data-sets (from here on referred to as “signal intensity”) as a measure for expression levels from the individual TSSs, and found a high correlation between the biological replicates, especially when the signal intensity was higher than five (Pearson coefficients >0.9) (Additional file 6: Figure S2A to D).

Finally, a closer look at the promoter sequences in the three additional organisms revealed a similar pattern to what was seen in the analyses of *S. aureus* TSS-EMOTE data. Logo-plots of the promoter sequences exhibited clear -10 elements for σ^A^/σ^70^ recognition and preference for purines at the +1 position. Furthermore, pyrimidines are highly enriched at the -1 position, with T being more common in A + T rich *S. epidermidis* (Fig. 6b).

## Conclusions

A number of techniques have been developed over the years to map the 5’-ends of individual RNA species, and with the recent arrival of high-throughput sequencing, a few methods have emerged that simultaneously map the transcription start sites on a large scale. The TSS-EMOTE method presented here allows the identification of transcription start sites and promoters similar to the dRNA-seq method. The three main strengths of the method are the absolutely precise mapping of the TSSs (even if they are very close to each other), the possibility for a quantitative evaluation of each TSS position thanks to the specially designed barcodes, and finally that all needed equipment for the wet-lab protocol can be found in a standard laboratory, all enzymes are available from standard suppliers, and each RNA sample only costs about $200 to prepare and sequence. The method can easily be applied to different bacteria and, because it allows quantification of transcription start sites via the UMIs, it is an ideal tool to compare different growth conditions of a given bacterium. It is our conviction that the data presented here will be highly useful for the scientific communities that study *S. aureus*, *S. epidermidis*, *A. baumannii* and *E. aerogenes*. In addition, the TSSs and operons defined by TSS-EMOTE will be highly useful as training sets for large-scale *in silico* operon prediction efforts such as the DOOR^2^ Database of prOkaryotic OpeRons, where experimental input data improves accuracy significantly [10, 49]. As a consequence of the above-mentioned advantages of TSS-EMOTE, we expect that in the near future, many prokaryotes as well as eukaryotes will be examined by TSS-EMOTE to answer the many biological questions that TSS analyses can illuminate.

## Methods

### Bacterial growth conditions and RNA isolation

RNA was isolated from exponentially growing cultures of *Staphylococcus aureus* MW2 (kindly provided by Dr. William Kelley, University of Geneva, Switzerland), *Staphylococcus epidermidis* ATCC 12228 (kindly provided by Dr. Arnaud Riat, Geneva University Hospital, Switzerland), *Enterobacter aerogenes* KCTC 2190 (kindly provided by Dr. Thilo Köhler, University of Geneva, Switzerland), and *Acinetobacter baumannii* ATCC 17978 (kindly provided by Prof. Gottfried Wilharm, Robert Koch Institute, Berlin, Germany) in the following manner.

Over-night cultures of *E. aerogenes* and *A. baumannii* were diluted 1:100 in LB medium (Merck) and the cultures were agitated at 37 °C until OD_600_ reached 0.5, whereupon 4 ml of the culture was added to 20 ml ice-cold ethanol/acetone (1:1 mix), which immediately kills the cells and inactivates all enzymes. The cells were pelleted by 5 min of 4000 g centrifugation at 4 °C, the supernatant removed, and 1 ml ethanol/acetone was added before the pellet was stored at -80 °C. To prepare for lysis, the pellet was centrifuged again at 4 °C for 5 min at 4000 g, and the ethanol/acetone supernatant removed. The pellet was then resuspended in 1 ml 1xTE buffer (10 mM Tris-HCl pH 8, 1 mM EDTA), transferred to a 1.5 ml microtube and re-pelleted by 2 min of 17000 g centrifugation. The cells were lysed in 100 μl TE buffer for 10 min at 37 °C with 100 μg lysozyme and 40 U RNasin Plus RNase A inhibitor (Promega), and pure RNA was immediately isolated using the ReliaPrep RNA Tissue Miniprep System (Promega).

An over-night culture of *S. aureus* MW2 was diluted 1:100 in RPMI medium (RPMI1640 with HEPES buffer, from Sigma-Aldrich R7388) or MH (cation adjusted Mueller-Hinton, from Becton Dickinson), or 50 μl was spread on an MH-agar plate (13 % agar). One set of MH cultures were agitated at 30 °C (MH30) and at OD_600_ = 0.4 harvested as described above, the remaining were incubated at 37 °C and either harvested at OD_600_ = 0.4 (MH37 and RPMI) or after 24 h (MH_Agar). The lawn of MH_Agar cells were scraped off the plate and plunged into ice cold ethanol/acetone. The RNA was isolated as for *E. aerogenes* and *A. baumannii,* but lysed with 100 μg lysostaphein instead of lysozyme. *S. epidermidis* was treated identically to the *S. aureus* MH37 culture as described above.

### TSS-EMOTE protocol

The TSS-EMOTE protocol is a further development of the original EMOTE protocol, which was designed to detect mono-phosphorylated 5’-ends, and details about the rationale behind the various oligo designs can be found in [18]. All water used in this protocol is molecular biology grade RNase-Free water (Amimed, Bioconcept, Allschwil, Switzerland). Micro tubes are also RNase-Free (Treff, Degersheim, Switzerland), as are the filtered micro-pipette tips used (Biotix, VWR International, Nyon, Switzerland and Sarstedt, Nümbrecht, Germany).

#### XRN1 digestion

10 μg total RNA from each sample was treated with 5 U XRN-1 (New England Biolabs) in a 80 μl volume of buffer NEB3, with 80 U RNasin Plus RNase inhibitor. After 4 h of incubation at 37 °C, 220 μl water was added and the enzymes were removed with two sequential phenol/chloroform/isoamyl (Sigma) extractions, using Phase Lock Gel (www.5prime.com) to facilitate separation of the phases. 20 μg glycogen (Roche) was added, and the salt concentration adjusted to 0.3 M sodium acetate, whereupon 3 volumes of 96 % ethanol were added. After thorough vortexing, the samples were stored over night at -80 °C, pelleted at 17000 g for 45 min at 4 °C, and washed twice in 750 μl cold 75 % ethanol. Finally, the XRN1 digested RNA was resuspended in 20 μl 0.1x TE buffer.

#### Ligation step

Two tubes for each RNA sample were prepared with 5 μl XRN-treated RNA and 50 pmol Rp6 oligo (Table 2). The tubes are heated to 70 °C for 5 min and then immediately flash-cooled in icewater. To each tube was added 10 μl of either “-RppH mix” or “+RppH mix”, which were then incubated at 37 °C. The “-RppH mix” consisted of 3.5 μl water, 2 μl RNA ligase buffer, 2 μl 10 mM ATP, 1 μl Murine RNase inhibitor (New England Biolabs), and 2 μl RNA ligase 1 (New England Biolabs). The “+RppH mix” consisted of 1.5 μl water, 2 ul RNA ligase buffer, 2 μl 10 mM ATP, 1 μl Murine RNase inhibitor, 2 μl RNA ligase 1, and 2 μl RppH (New England Biolabs). After 30 min of incubation, 1 μl 10 mM ATP was added, and the reaction was incubated over night at 16 °C. 70 μl water, 10 μl 3 M sodium acetate and 0.5 μl glycogen (20 μg/μl) was added and the tubes were vortexed. Then 300 μl ethanol was added, and the tubes were vortexed again, to precipitate the ligated RNA over night at -80 °C. The ligated RNA was pelleted for 45 min at 17000 g and 4 °C, then washed once with 900 μl cold 75 % ethanol, and resuspended in 20 μl 0.1xTE. The samples were heated to 70 °C for 10 min to dissolve RNA well, and to inactivate any residual enzymatic activity.

**Table 2:** Oligos

Name	Sequence
Rp6	CGGCACCAACCGAGGVVVVVVVCGC (RNA)
DROAA	GGCATTCCTGCTGAACCGCTCTTCCGATCTNNNNNNNNAA
D6A:	CTCTTTCCCTACACGACGCTCTTCCGATCTNTACACGGCACCAACCGAGG
D6B:	CTCTTTCCCTACACGACGCTCTTCCGATCTNGTATCGGCACCAACCGAGG
D6C:	CTCTTTCCCTACACGACGCTCTTCCGATCTNCGTCCGGCACCAACCGAGG
D6D:	CTCTTTCCCTACACGACGCTCTTCCGATCTNAAGTCGGCACCAACCGAGG
D6E:	CTCTTTCCCTACACGACGCTCTTCCGATCTNACACCGGCACCAACCGAGG
D6F:	CTCTTTCCCTACACGACGCTCTTCCGATCTNGGTACGGCACCAACCGAGG
D6H:	CTCTTTCCCTACACGACGCTCTTCCGATCTNTCGGCGGCACCAACCGAGG
D6I:	CTCTTTCCCTACACGACGCTCTTCCGATCTNCAAGCGGCACCAACCGAGG
D6J:	CTCTTTCCCTACACGACGCTCTTCCGATCTNTTGACGGCACCAACCGAGG
D6K:	CTCTTTCCCTACACGACGCTCTTCCGATCTNGCTGCGGCACCAACCGAGG
D6L:	CTCTTTCCCTACACGACGCTCTTCCGATCTNCCGACGGCACCAACCGAGG
D6M:	CTCTTTCCCTACACGACGCTCTTCCGATCTNCTCGCGGCACCAACCGAGG
D6N:	CTCTTTCCCTACACGACGCTCTTCCGATCTNAGGACGGCACCAACCGAGG
D6O:	CTCTTTCCCTACACGACGCTCTTCCGATCTNATTGCGGCACCAACCGAGG
D6P:	CTCTTTCCCTACACGACGCTCTTCCGATCTNGACGCGGCACCAACCGAGG
D6Q:	CTCTTTCCCTACACGACGCTCTTCCGATCTNTGTTCGGCACCAACCGAGG
A-PE-PCR10	AATGATACGGCGACCACCGAGATCTACACTCTTTCCCTACACGACG
B-PE-PCR20	CAAGCAGAAGACGGCATACGAGATCGGTCTCGGCATTCCTGCTGAACCGC

#### Reverse transcription

The DROAA oligo (Table 2) used to prime the formation of cDNA is designed to hybridise almost completely randomly on the RNA, however two A’s have been added at the extreme 3’-end of the DROAA oligo to reduce the number of possible priming sites approximate 16 times (it varies according to the G + C content of the organism). This semi-random design furthermore serves to prevent the DROAA from priming on the Rp6 oligo (which contains no uridine residues), which is present in excess at this step of the protocol.

For each ligation reaction, 8 μl Rp6-ligated RNA, was mixed with 1 μl 100 mM DTT and 1 μl 20 μM DROAA oligo, then heated to 75 °C for 3 min and the tubes were allowed to cool slowly to room temperature. An RT-mix was prepared for each tube, with 3 μl water, 4 μl 5x Reverse Transcriptase buffer (New England Biolabs), 1 μl 100 mM DTT, 1 μl 10 mM dNTP, 0.5 μl Murine RNase Inhibitor Murine and 1 μl M-MLV (-H) Reverse Transcriptase (New England Biolabs). 10 μl of the RT-mix was added to each tube of RNA + primer, which was incubated 10 min at room temperature, then 50 min at 42 °C, and finally 30 min at 65 °C (to inactivate enzymes). Finally, the cDNA was purified using a PCR-purification kit (GeneJET Gel Extraction Kit, Thermo Scientific, Milian, Vernier, Switzerland), and eluted in 50 μl Elution Buffer.

#### Second-strand PCR

To generate double stranded DNA, with the appropriate adaptors for Illumina sequencing, as well as addition of a four nucleotide EMOTE barcode which serves to identify the RNA sample and pool, a PCR reaction was prepared for each RT-reaction: 10 μl PCR-purified RT-reaction, 27 μl water, 10 μl Q5 Polymerase buffer, 1.5 μl dNTP (2.5 mM each), 1.5 μl 100nM of one of primers D6A, D6B, D6C, etc. (each with a unique EMOTE barcode, Table 2), 1.5 μl 10 uM Primer B-PE-PCR20, and 0.5 μl Q5® Hot Start High-Fidelity DNA Polymerase (NEB).

These 50 μl PCR reactions with low D6x primer concentration was run for the 5 first cycles of the following program: 2 min @98 °C, (10s @98 °C, 20s @50 °C, 2 min @ 72 °C) 31 cycles, 5 min @72 °C and finally 4 °C. At the end of cycle 5, the PCR machine was paused, and 1.5 μl 10 μM A-PE-PCR10 was added (Table 2), and the tubes replaced in the PCR machine for the program to continue for another 25 cycles. To visually verify that the yields were similar, 10 μl of the PCR reactions was loaded on an agarose gel, and the remaining 40 μl from each PCR were mixed with 40 μl Binding Buffer (GeneJET Gel Extraction Kit) and 20 μl Isopropanol, to be purified according to protocol and eluted in 40 μl elution buffer.

#### Size-selection of PCR-products

The Illumina technology gives poor results with inserts above 800 bp, and the TSS-EMOTE protocol frequently yields low molecular weight products (probably a mixture of unincorporated primers, false priming products and Rp6-concatamers). A 1 % agarose gel was therefore used to select PCR-products in the size-range 300 bp to 1000 bp, which were subsequently extracted from the gel with GeneJET Gel Extraction Kit, adding an equal volume of isopropanol during binding (to ensure that DNA below 500 bp was not lost), and eluted in 50 μl Elution Buffer. The upper limit in PCR-product length should not bias the TSS-EMOTE assay towards shorter transcripts, since the Revers Transcription reaction is based on random priming of the DROAA oligo. Thus each transcript will give rise to a random range of cDNA molecules, all ending in a sequence complementary to the Rp6 oligo, but with different start positions and therefore different lengths.

### Illumina sequencing

The concentration of the DNA recovered from the agarose gel (see above) were quantified with a Qubit Fluorometer, diluted appropriately, and loaded onto a HiSeq 2500 machine (Illumina, San Diego, USA). Between 6 and 14 million reads were obtained for each RNA sample, and on average about 3 million of these could be mapped onto their respective genomes.

Additionally, the Illumina TruSeq total RNA stranded protocol was used to prepare a library of the same RNA that was used for the TSS-EMOTE of RNA from *S. aureus* grown in RPMI medium. The resulting 100 nt paired-end reads were mapped to the reference genome with Bowtie [50]. However, for further analyses (for example in Figs. 2d and 4), only the upstream reads were counted in order to maximise the signal near the 5’-ends.

### *In silico* TSS identification

The raw sequence read data from the Illumina sequencing was converted to EMOTE table-format according to the principles described in [18], using the EMOTE-conv software package [51], which lists all genomic positions for which an RNA 5'-end has been detected, with the number of Illumina reads and the number of Unique Molecular Identifiers (UMIs) (i.e. *bona fide* unique ligation events) that correspond to each position (examples of read-counts plotted against UMIs are shown in Additional file 7: Figure S3). With the Rp6 oligo used in this study, the maximum UMI-count for a given position is 2187 (=37). This value was not reached in the data presented here, however if saturation becomes a problem in future projects, then the EMOTE-conv software permits down-sampling of the Illumina fastq-file, to get below saturation level.

The data in the EMOTE tables were then analysed as described in the results section to identify the TSSs. The reference sequences used were: NC_003923 (*S. aureus* MW2 chromosome), CP000521 (*Acinetobacter baumannii* ATCC 17978 chromosome), CP000522 (*Acinetobacter baumannii* ATCC 17978 plasmid pAB1), CP000523 (*Acinetobacter baumannii* ATCC 17978 plasmid pAB2), CP012004.1 (*Acinetobacter baumannii* ATCC 17978-mff chromosome), CP012005.1 (*Acinetobacter baumannii* ATCC 17978-mff plasmid pAB3), NC_004461 (*Staphylococcus epidermidis* ATCC 12228 chromosome), NC_005008 (*Staphylococcus epidermidis* ATCC 12228 plasmid pSE-12228-01), NC_005007 (*Staphylococcus epidermidis* ATCC 12228 plasmid pSE-12228-02), NC_005006 (*Staphylococcus epidermidis* ATCC 12228 plasmid pSE-12228-03), NC_005005 (*Staphylococcus epidermidis* ATCC 12228 plasmid pSE-12228-04), NC_005004 (*Staphylococcus epidermidis* ATCC 12228 plasmid pSE-12228-05), NC_005003 (*Staphylococcus epidermidis* ATCC 12228 plasmid pSE-12228-06), and NC_015663 (*Enterobacter aerogenes* KCTC 2190 chromosome).

A number of statistical tools have been developed for mRNA-seq differential expression analysis [52, 53], however they are designed to compare mRNA levels of two separate RNA samples (each sample representing multiple biological replicates). In contrast, in TSS-EMOTE data, the comparison is within the same RNA sample, only treated with slightly different enzyme mixtures. Therefore, we favour a simple statistical test that rely on a beta-binomial distribution to integrate this information, and is able to assign a p-value to each potential TSS:

Let N^+^ and N^-^ be the number of reads that align onto the genome in the + RppH and the -RppH pools. Let also q^+^ and q^-^ be the UMIs obtained for a given position on the genome in the two pools. Our model assumes that in absence of TSS, q^+^ follows a beta-binomial distribution parameterized by q^-^, N^-^ and N^+^ as follows,

$$ P\left({q}^{+}\left|{N}^{+},\kern0.5em {q}^{-},\kern0.5em {N}^{-}\right.\right)\kern0.5em =\kern0.5em \beta \_ binomial\begin{array}{c}\hfill \beta =1+{N}^{-}-{q}^{-}\hfill \\ {}\hfill a=1+{q}^{-}\kern3.5em \hfill \end{array}\left({q}^{+},\kern0.5em {N}^{+}\right). $$

The area under the upper tail of the distribution reflects how the data deviate from the model, and reveal our confidence in the position to be a TSS. Once the model for each -RppH/+RppH pair has been calculated, p-values of biological replicates of the TSS-EMOTE assay are further combined with Fisher’s method. Probabilities are adjusted for multiple testing by computing the False Discovery Rate (FDR), and requiring TSS sites to have a FDR < 0.01. Only candidates with q^+^ ≥ 5 reads in one of the replicates are considered in this TSS detection process. An example of the UMI-corrected read-counts mapping to the *sarA* locus of *S. aureus* are shown in Additional file 8: Figure S4B, together with the TSSs previously identified by others (Additional file 1: Table S1) and the TSSs identified in this study (Additional file 3: Table S2).

All computations are performed with the R programming language, making use of VGAM package for the beta binomial distribution [54, 55], and the R-scripts used are available upon request.

### TSS annotation

Identified TSSs further enter an annotation process where we determine: 1) if the TSS fall inside an annotated ORF; 2) the name of the first annotated ORF following the TSSs position in a strand specific manner; 3) the 50 bp surrounding sequence (between positions -45 and +5 around the TSS), into which we look for a match of the sigma-factor A recognition pattern TATAAT and TTGACA around positions -11 and -37 respectively. Additionally, TSSs that are less than 5 bp apart from each other are clustered into a TSS cluster, and the TSS with the smallest p-value is considered as the representative TSS (rTSS) of this cluster.

Finally, *S. aureus* TSSs are annotated with the closest predicted non-coding RNA for MW2 strain taken from the staphylococcal regulatory RNA database [22]. We also make use of the work of [4] on the sigma-factor B regulon to flag rTSSs that immediately precede an ORF upregulated by sigma-factor B in *S. aureus*.

### Transcription terminator prediction

Transcription terminators are predicted using the software TransTermHP v2.09 [39]. The program is run with standard parameter values, and an annotation file containing coordinates of all annotated genes.

### Sequence logos

All sequence logos were generated with the R package motifStack from Bioconductor, and using the background correction to adjust the signal according to GC content of the organism [56].

## Additional files

Additional file 1: Table S1. Re-detection of previously identified TSSs. (XLSX 13 kb)
Additional file 2: Figure S1. Coverage plot with traces for all rTSSs. The 2018 black lines show the RNA-seq coverage profile around all 2018 rTSSs detected in *S. aureus* MW2 grown in RPMI medium. The bold blue line is the median profile (also shown in Fig. 4), and the dotted lines correspond to the 25th and 75th percentiles (also shown in Fig. 4). The vertical dotted line marks the +1 position. (PDF 15627 kb)
Additional file 3: Table S2. List of identified TSSs. Note that this table includes two sheets for *A. baumannii*, corresponding to two published reference genomes (ATCC 17978, and ATCC 17978-mff). (XLSX 3099 kb)
Additional file 4: Table S3. TSS clusters with six or more TSSs. (DOCX 15 kb)
Additional file 5: Table S4. Predicted operon structure based on TSS-EMOTE experimental data and *in silico* transcription terminator finding. Note that this table includes two sheets for *A. baumannii*, corresponding to two published reference genomes (ATCC 17978, and ATCC 17978-mff). (XLSX 1528 kb)
Additional file 6: Figure S2. Correlation in signal intensity (+RppH UMI-values) between biological replicates. The x-axis and the y-axis are biological replicate 1 and 2, respectively. All values of zero were set to one, in order to avoid the mathematical impossibility of plotting zero into a logarithmic plot. The Pearsson-coefficients given are based on data-points higher than 5 for both replicates (above the dotted lines). (PDF 16518 kb)
Additional file 7: Figure S3. Correlation between raw read-counts and UMI-value. Each dot corresponds to a detected TSS in the data from *S. aureus* grown in RPMI medium at 37 °C. The theoretical upper limit of the UMIs is 3^7^ = 2187, which shown as a horizontal dotted line (the Rp6 oligo contains 7 nucleotides that are randomly chosen among A, C or G). The diagonal is indicated with a solid line. (PDF 5917 kb)
Additional file 8: Figure S4. Screenshots demonstrating how the TSS-EMOTE reads map in the *sarA* region. A) The mapping sequences (the 20 nt after the Control Sequence) from the TSS-EMOTE data for *S. aureus* grown in RPMI medium were mapped onto the *sarA* locus (which has three previously identified TSSs, see also Additional file 1: Table S1). Note that in this screen-shot, regions with high coverage have many reads that are outside the “window” (examples marked with blue circles). However, the true coverage can be seen as dark grey columns (examples indicated by red arrows). B) Same data as with panel A, but using the UMI-corrected read-counts (meaning that if several reads that map to the same position have identical UMIs, then only a single read is used). Only the first nucleotides of the mapping-sequences are shown, and these represent the 5’ nucleotides of the original RNA molecules. TSSs previously identified by others (see also Additional file 1: Table S1) and the TSSs identified in this study (see also Additional file 3: Table S2) are also shown (bottom part of the figure). (PDF 624 kb)

## Abbreviations

FDR: False discovery rate
MH: Mueller-Hinton medium
ORF: Open reading frame
RBS: Ribosome binding site
rTSS: Representative transcription start site
TSS: Transcription start site
TSS-EMOTE: Transcription start specific exact mapping of transcriptome ends
UMI: Unique molecular identifier

## References

[1] Feklístov A, Sharon BD, Darst SA, Gross CA (2014). Bacterial sigma factors: a historical, structural, and genomic perspective. Annu Rev Microbiol.

[2] Morikawa K, Inose Y, Okamura H, Maruyama A, Hayashi H, Takeyasu K (2003). A new staphylococcal sigma factor in the conserved gene cassette: functional significance and implication for the evolutionary processes. Genes Cells.

[3] Shaw LN, Lindholm C, Prajsnar TK, Miller HK, Brown MC, Golonka E (2008). Identification and characterization of sigma, a novel component of the *Staphylococcus aureus stress* and virulence responses. PLoS One.

[4] Bischoff M, Dunman P, Kormanec J, Macapagal D, Murphy E, Mounts W (2004). Microarray-based analysis of the *Staphylococcus aureus* sigmaB regulon. J Bacteriol.

[5] Pané-Farré J, Jonas B, Förstner K, Engelmann S, Hecker M (2006). The sigmaB regulon in *Staphylococcus aureus* and its regulation. Int J Med Microbiol.

[6] Murakami KS, Darst SA (2003). Bacterial RNA polymerases: the wholo story. Curr Opin Struct Biol.

[7] Vvedenskaya IO, Zhang Y, Goldman SR, Valenti A, Visone V, Taylor DM (2015). Massively systematic transcript end readout, “MASTER”: transcription start site selection, transcriptional slippage, and transcript yields. Mol Cell.

[8] Robb NC, Cordes T, Hwang LC, Gryte K, Duchi D, Craggs TD (2013). The transcription bubble of the RNA polymerase-promoter open complex exhibits conformational heterogeneity and millisecond-scale dynamics: implications for transcription start-site selection. J Mol Biol.

[9] Jacob F, Monod J (1961). Genetic regulatory mechanisms in the synthesis of proteins. J Mol Biol.

[10] Dam P, Olman V, Harris K, Su Z, Xu Y (2007). Operon prediction using both genome-specific and general genomic information. Nucleic Acids Res.

[11] ten Broeke-Smits NJP, Pronk TE, Jongerius I, Bruning O, Wittink FR, Breit TM (2010). Operon structure of *Staphylococcus aureus*. Nucleic Acids Res.

[12] Ruiz de los Mozos I, Vergara-Irigaray M, Segura V, Villanueva M, Bitarte N, Saramago M (2013). Base pairing interaction between 5’- and 3’-UTRs controls icaR mRNA translation in *Staphylococcus aureus*. PLoS Genet.

[13] Oliva G, Sahr T, Buchrieser C (2015). Small RNAs, 5’ UTR elements and RNA-binding proteins in intracellular bacteria: impact on metabolism and virulence. FEMS Microbiol Rev.

[14] Sharma CM, Vogel J (2014). Differential RNA-seq: the approach behind and the biological insight gained. Curr Opin Microbiol.

[15] Baba T, Takeuchi F, Kuroda M, Yuzawa H, Aoki K, Oguchi A (2002). Genome and virulence determinants of high virulence community-acquired MRSA. Lancet.

[16] Deana A, Celesnik H, Belasco JG (2008). The bacterial enzyme RppH triggers messenger RNA degradation by 5’ pyrophosphate removal. Nature.

[17] Song M-G, Bail S, Kiledjian M (2013). Multiple Nudix family proteins possess mRNA decapping activity. RNA.

[18] Redder P (2015). Using EMOTE to map the exact 5’-ends of processed RNA on a transcriptome-wide scale. Methods Mol Biol.

[19] Hawley DK, McClure WR (1983). Compilation and analysis of *Escherichia coli* promoter DNA sequences. Nucleic Acids Res.

[20] Petersohn A, Bernhardt J, Gerth U, Höper D, Koburger T, Völker U (1999). Identification of sigma(B)-dependent genes in *Bacillus subtilis* using a promoter consensus-directed search and oligonucleotide hybridization. J Bacteriol.

[21] Lasa I, Toledo-Arana A, Dobin A, Villanueva M, de los Mozos IR, Vergara-Irigaray M (2011). Genome-wide antisense transcription drives mRNA processing in bacteria. Proc Natl Acad Sci USA.

[22] Sassi M, Augagneur Y, Mauro T, Ivain L, Chabelskaya S, Hallier M (2015). SRD: a *Staphylococcus* regulatory RNA database. RNA.

[23] Khemici V, Prados J, Linder P, Redder P (2015). Decay-initiating endoribonucleolytic cleavage by RNase Y is kept under tight control via sequence preference and sub-cellular localisation. PLoS Genet.

[24] Kirkpatrick CL, Martins D, Redder P, Frandi A, Mignolet J, Chapalay JB (2016). Growth control switch by a DNA-damage-inducible toxin–antitoxin system in *Caulobacter crescentus*. Nat Microbiol.

[25] Rao L, Karls RK, Betley MJ (1995). In vitro transcription of pathogenesis-related genes by purified RNA polymerase from *Staphylococcus aureus*. J Bacteriol.

[26] Bayer MG, Heinrichs JH, Cheung AL (1996). The molecular architecture of the sar locus in *Staphylococcus aureus*. J Bacteriol.

[27] Steinhuber A, Goerke C, Bayer MG, Döring G, Wolz C (2003). Molecular architecture of the regulatory Locus sae of *Staphylococcus aureus* and its impact on expression of virulence factors. J Bacteriol.

[28] Rice KC, Patton T, Yang S-J, Dumoulin A, Bischoff M, Bayles KW (2004). Transcription of the *Staphylococcus aureus* cid and lrg murein hydrolase regulators is affected by sigma factor B. J Bacteriol.

[29] Gao J, Stewart GC (2004). Regulatory elements of the *Staphylococcus aureus* protein A (Spa) promoter. J Bacteriol.

[30] Koprivnjak T, Mlakar V, Swanson L, Fournier B, Peschel A, Weiss JP (2006). Cation-induced transcriptional regulation of the dlt operon of *Staphylococcus aureus*. J Bacteriol.

[31] Harraghy N, Homerova D, Herrmann M, Kormanec J (2008). Mapping the transcription start points of the *Staphylococcus aureus eap*, *emp*, and *vwb* promoters reveals a conserved octanucleotide sequence that is essential for expression of these genes. J Bacteriol.

[32] Hsieh H-Y, Tseng CW, Stewart GC (2008). Regulation of Rot expression in *Staphylococcus aureus*. J Bacteriol.

[33] Geissmann T, Chevalier C, Cros M-J, Boisset S, Fechter P, Noirot C (2009). A search for small noncoding RNAs in *Staphylococcus aureus* reveals a conserved sequence motif for regulation. Nucleic Acids Res.

[34] Nygaard TK, Pallister KB, Ruzevich P, Griffith S, Vuong C, Voyich JM (2010). SaeR binds a consensus sequence within virulence gene promoters to advance USA300 pathogenesis. J Infect Dis.

[35] Bohn C, Rigoulay C, Chabelskaya S, Sharma CM, Marchais A, Skorski P (2010). Experimental discovery of small RNAs in *Staphylococcus aureus* reveals a riboregulator of central metabolism. Nucleic Acids Res.

[36] Jeong D-W, Cho H, Lee H, Li C, Garza J, Fried M (2011). Identification of the P3 promoter and distinct roles of the two promoters of the SaeRS two-component system in *Staphylococcus aureus*. J Bacteriol.

[37] Weiss A, Ibarra JA, Paoletti J, Carroll RK, Shaw LN (2014). The δ subunit of RNA polymerase guides promoter selectivity and virulence in *Staphylococcus aureus*. Infect Immun.

[38] Cheung GYC, Joo H-S, Chatterjee SS, Otto M (2014). Phenol-soluble modulins--critical determinants of staphylococcal virulence. FEMS Microbiol Rev.

[39] Kingsford CL, Ayanbule K, Salzberg SL (2007). Rapid, accurate, computational discovery of Rho-independent transcription terminators illuminates their relationship to DNA uptake. Genome Biol.

[40] Jousselin A, Kelley WL, Barras C, Lew DP, Renzoni A (2013). The *Staphylococcus aureus* thiol/oxidative stress global regulator Spx controls trfA, a gene implicated in cell wall antibiotic resistance. Antimicrob Agents Chemother.

[41] Zhang Y-Q, Ren S-X, Li H-L, Wang Y-X, Fu G, Yang J (2003). Genome-based analysis of virulence genes in a non-biofilm-forming *Staphylococcus epidermidis* strain (ATCC 12228). Mol Microbiol.

[42] Smith MG, Gianoulis TA, Pukatzki S, Mekalanos JJ, Ornston LN, Gerstein M (2007). New insights into *Acinetobacter baumannii* pathogenesis revealed by high-density pyrosequencing and transposon mutagenesis. Genes Dev.

[43] Rice LB (2008). Federal funding for the study of antimicrobial resistance in nosocomial pathogens: no ESKAPE. J Infect Dis.

[44] Boucher HW, Talbot GH, Bradley JS, Edwards JE, Gilbert D, Rice LB (2009). Bad bugs, no drugs: no ESKAPE! An update from the Infectious Diseases Society of America. Clin Infect Dis.

[45] Shin SH, Kim S, Kim JY, Lee S, Um Y, Oh M-K (2012). Complete genome sequence of *Enterobacter aerogenes* KCTC 2190. J Bacteriol.

[46] Diene SM, Merhej V, Henry M, El Filali A, Roux V, Robert C (2013). The rhizome of the multidrug-resistant *Enterobacter aerogenes* genome reveals how new “killer bugs” are created because of a sympatric lifestyle. Mol Biol Evol.

[47] Pendleton JN, Gorman SP, Gilmore BF (2013). Clinical relevance of the ESKAPE pathogens. Expert Rev Anti Infect Ther.

[48] Linder P, Lemeille S, Redder P (2014). Transcriptome-wide analyses of 5’-ends in RNase J mutants of a gram-positive pathogen reveal a role in RNA maturation, regulation and degradation. PLoS Genet.

[49] Mao F, Dam P, Chou J, Olman V, Xu Y (2009). DOOR: a database for prokaryotic operons. Nucleic Acids Res.

[50] Langmead B, Trapnell C, Pop M, Salzberg SL (2009). Ultrafast and memory-efficient alignment of short DNA sequences to the human genome. Genome Biol.

[51] Yasrebi H, Redder P. EMOTE-conv: a computational pipeline to convert exact mapping of transcriptome ends (EMOTE) data to the lists of quantified genomic positions correlated to related genomic information. J Appl Bioinform Comput Biol [Internet]. 2015 [cited 2016 Jan 27];4. Available from: http://scitechnol.com/emoteconv-a-computational-pipeline-to-convert-exact-mapping-of-transcriptome-ends-emote-data-to-the-lists-of-quantified-genomic-positions-correlated-to-related-genomic-information-vkxr.php?article_id=3708.

[52] Anders S, Huber W (2010). Differential expression analysis for sequence count data. Genome Biol.

[53] Robinson MD, McCarthy DJ, Smyth GK (2010). edgeR: a Bioconductor package for differential expression analysis of digital gene expression data. Bioinformatics.

[54] Yee TW. The VGAM package for categorical data analysis. J Stat Softw. 2010; 1(Issue 10) (2010) [Internet]; Available from: https://www.jstatsoft.org/index.php/jss/article/view/v032i10.

[55] R Core Team (2015). R: A Language and Environment for Statistical Computing [Internet].

[56] Huber W, Carey VJ, Gentleman R, Anders S, Carlson M, Carvalho BS (2015). Orchestrating high-throughput genomic analysis with Bioconductor. Nat Methods.

