# Supplementary figures and images for: TSS-EMOTE, a refined protocol for a more complete and less biased global mapping of transcription start sites in bacterial pathogens
Source: BMC Genomics. 2016 Nov 2;17:849. doi: 10.1186/s12864-016-3211-3 (PMC5094136; doi:10.1186/s12864-016-3211-3)

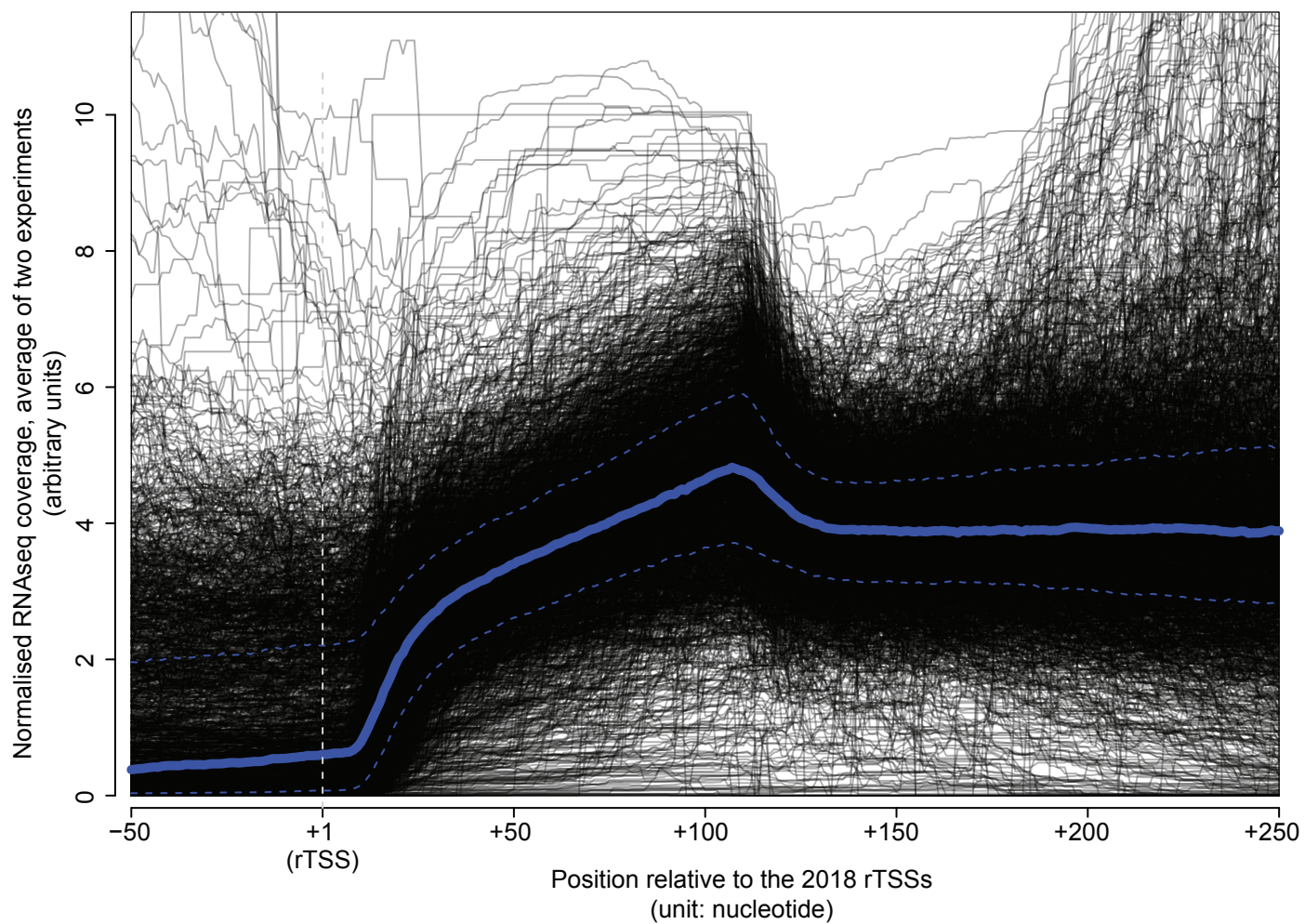

Supplement: Additional file 2: Figure S1. — Coverage plot with traces for all rTSSs. The 2018 black lines show the RNA-seq coverage profile around all 2018 rTSSs detected in S. aureus MW2 grown in RPMI medium. The bold blue line is the median profile (also shown in Fig. 4), and the dotted lines correspond to the 25th and 75th percentiles (also shown in Fig. 4). The vertical dotted line marks the +1 position. (PDF 15627 kb) [file 12864_2016_3211_MOESM2_ESM.pdf]

*S. aureus* RPMI

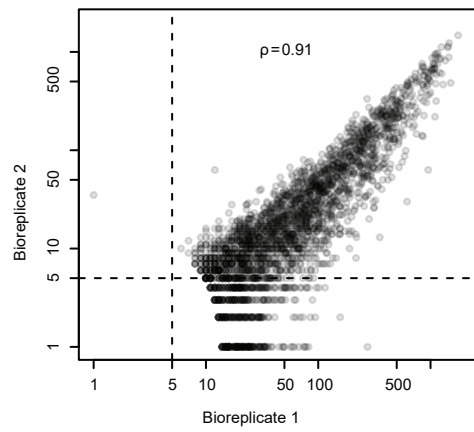

*S. epidermidis*

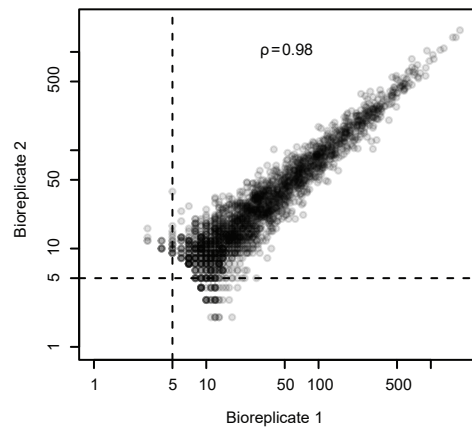

*A. baumannii*

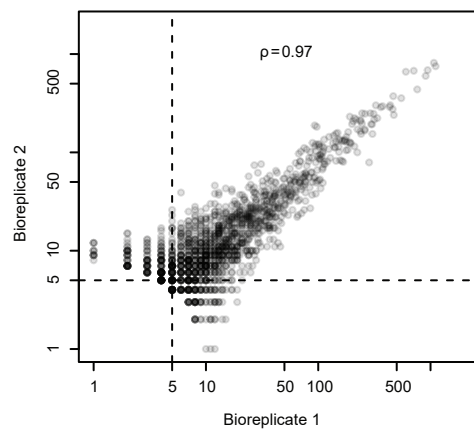

*E. aerogenes*

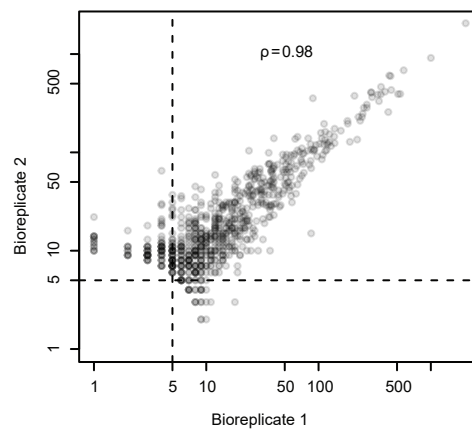

*S. aureus* MH\_agar 37°C

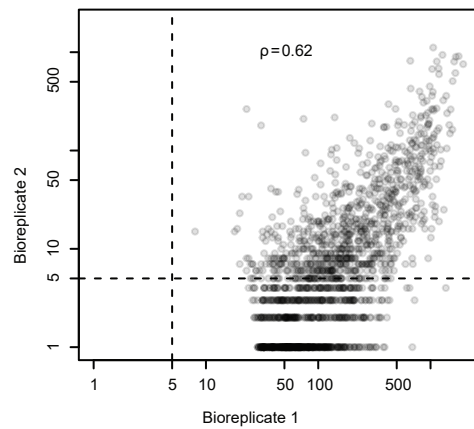

*S. aureus* MH 37°C

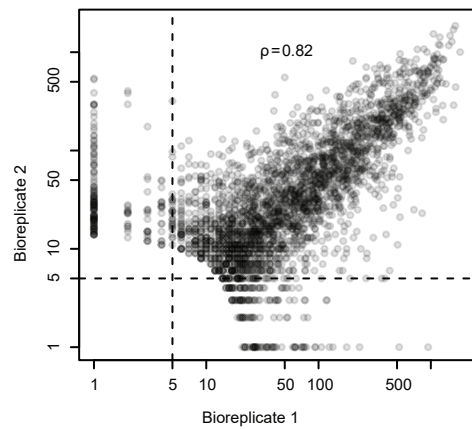

*S. aureus* MH 30°C

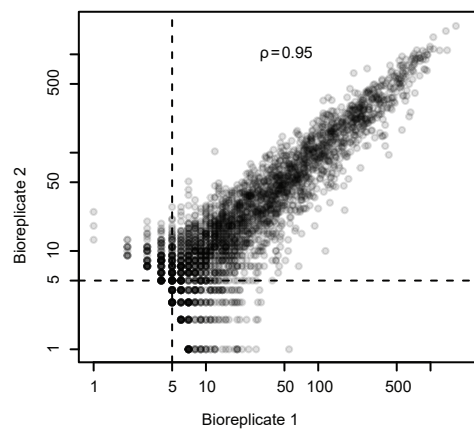

Supplement: Additional file 6: Figure S2. — Correlation in signal intensity (+RppH UMI-values) between biological replicates. The x-axis and the y-axis are biological replicate 1 and 2, respectively. All values of zero were set to one, in order to avoid the mathematical impossibility of plotting zero into a logarithmic plot. The Pearsson-coefficients given are based on data-points higher than 5 for both replicates (above the dotted lines). (PDF 16518 kb) [file 12864_2016_3211_MOESM6_ESM.pdf]

*S. aureus* MW2, RMPI medium 37°C

Bioreplicate 1

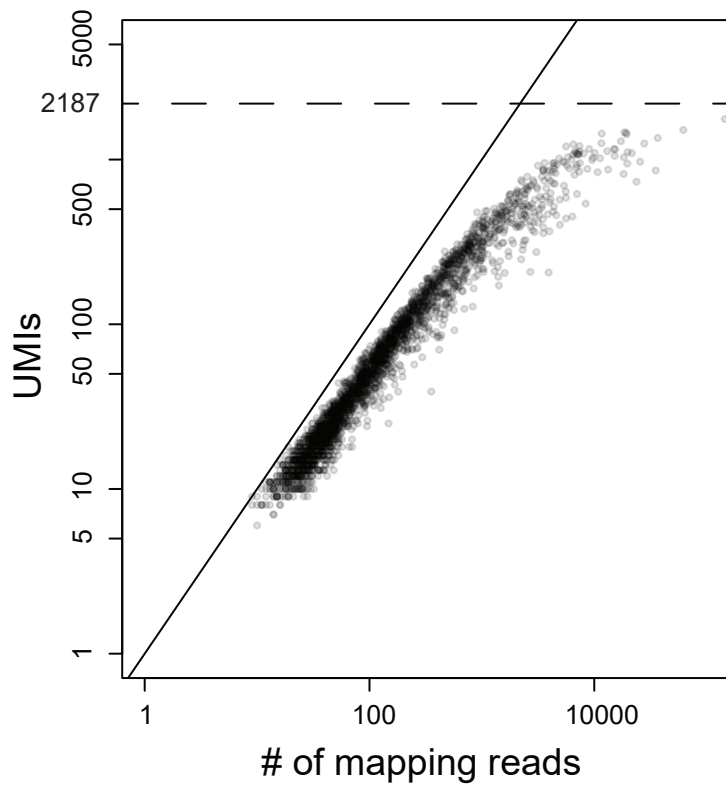

Bioreplicate 2

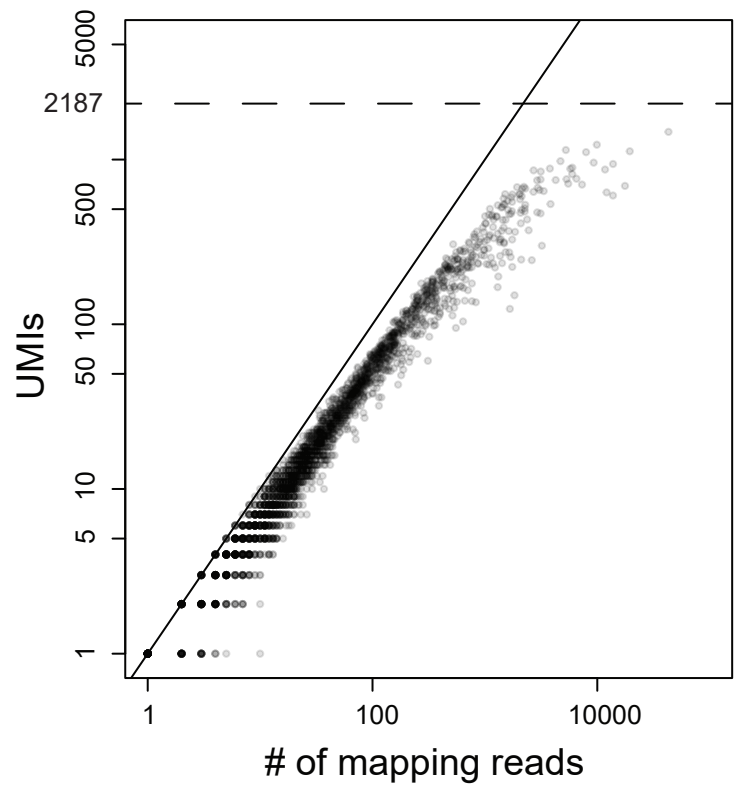

Supplement: Additional file 7: Figure S3. — Correlation between raw read-counts and UMI-value. Each dot corresponds to a detected TSS in the data from S. aureus grown in RPMI medium at 37 °C. The theoretical upper limit of the UMIs is 37 = 2187, which shown as a horizontal dotted line (the Rp6 oligo contains 7 nucleotides that are randomly chosen among A, C or G). The diagonal is indicated with a solid line. (PDF 5917 kb) [file 12864_2016_3211_MOESM7_ESM.pdf]

A

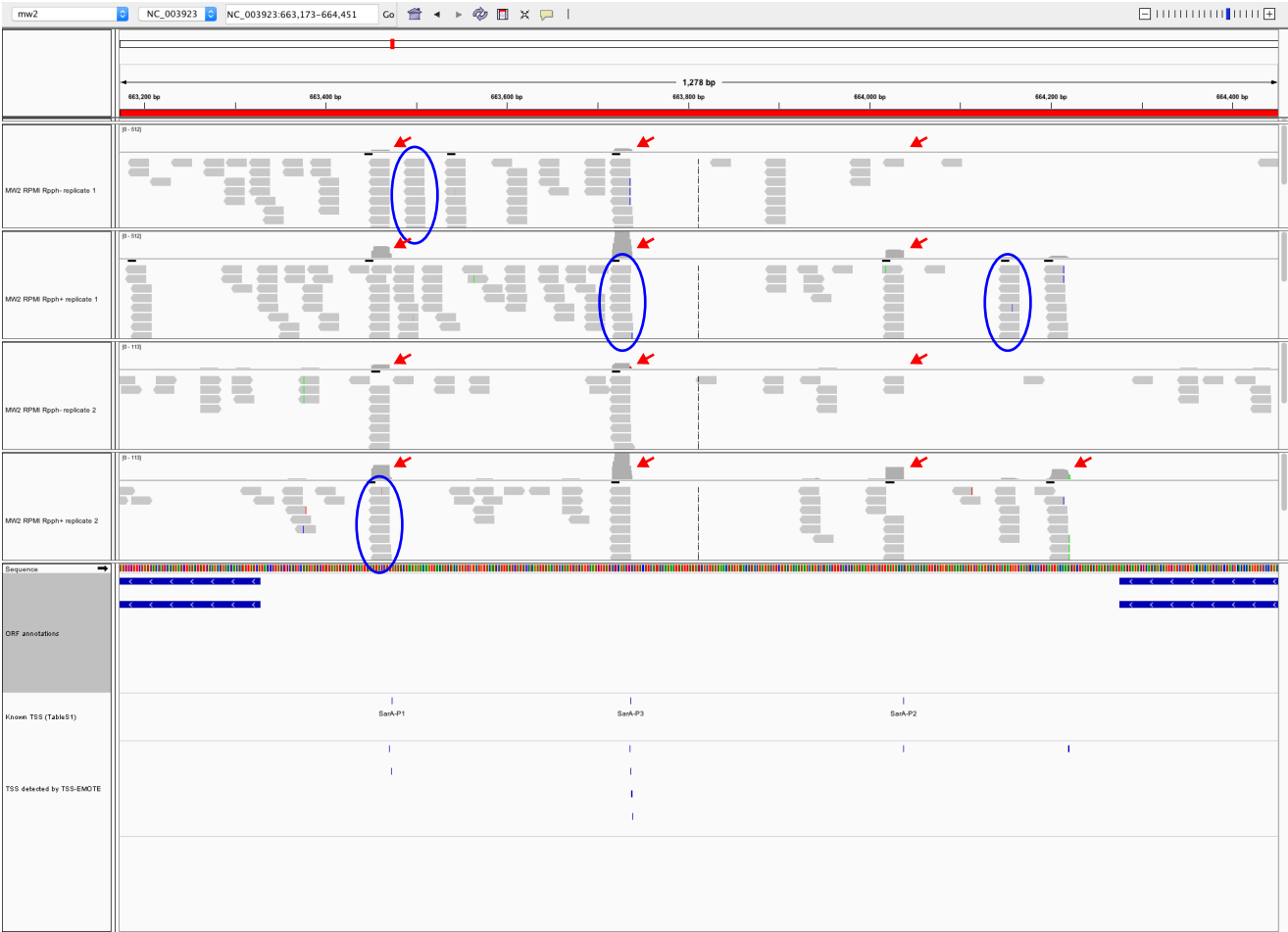

B

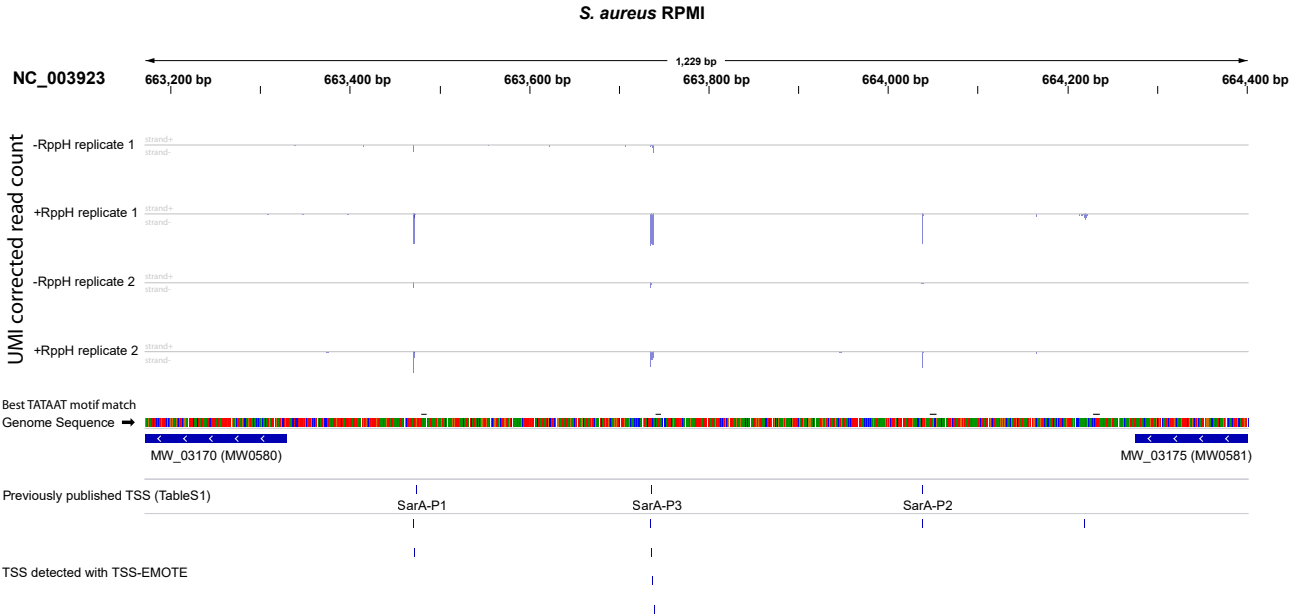

Supplement: Additional file 8: Figure S4. — Screenshots demonstrating how the TSS-EMOTE reads map in the sarA region. A) The mapping sequences (the 20 nt after the Control Sequence) from the TSS-EMOTE data for S. aureus grown in RPMI medium were mapped onto the sarA locus (which has three previously identified TSSs, see also Additional file 1: Table S1). Note that in this screen-shot, regions with high coverage have many reads that are outside the “window” (examples marked with blue circles). However, the true coverage can be seen as dark grey columns (examples indicated by red arrows). B) Same data as with panel A, but using the UMI-corrected read-counts (meaning that if several reads that map to the same position have identical UMIs, then only a single read is used). Only the first nucleotides of the mapping-sequences are shown, and these represent the 5’ nucleotides of the original RNA molecules. TSSs previously identified by others (see also Additional file 1: Table S1) and the TSSs identified in this study (see also Additional file 3: Table S2) are also shown (bottom part of the figure). (PDF 624 kb) [file 12864_2016_3211_MOESM8_ESM.pdf]
