# Supplementary material for: TSS-EMOTE, a refined protocol for a more complete and less biased global mapping of transcription start sites in bacterial pathogens
Source: BMC Genomics. 2016 Nov 2;17:849. doi: 10.1186/s12864-016-3211-3 (PMC5094136; doi:10.1186/s12864-016-3211-3)
Supplement: Additional file 4: Table S3. — TSS clusters with six or more TSSs. (DOCX 15 kb) [file 12864_2016_3211_MOESM4_ESM.docx]

Table S3, TSS clusters of five or more TSSs.

| rTSS position | Strand | Distance to downstream ORF | ID of downstream ORF | Comment | TSSs in cluster | Expected to be highly expressed | Overlapping σ^A^ and σ^B^ promoters (predicted) | Downstream gene is a Phenol Soluble Modulin | Unknown |
| --- | --- | --- | --- | --- | --- | --- | --- | --- | --- |
| 489708 | + | 51 | MW_RS02300 | Upstream of rRNA | 6 | X |  |  |  |
| 1105099 | + | 24 | MW_RS05415 | Hyp. prot. | 6 |  |  |  | X |
| 1152316 | + | 88 | MW_RS05690 | PSM β1 + β2 | 8 |  |  | X |  |
| 1152381 | + | 23 | MW_RS05690 | PSM β2* | 33 |  |  | X |  |
| 1165258 | + | 297 | MW_RS05755 | FtsZ, cell division | 6 |  |  |  | X |
| 1524247 | + | 448 | MW_RS07375 | Non-coding RNA sRNA243 (distance ~151 nt) | 8 |  |  |  | X |
| 903001 | - | 41 | MW_RS04430 | Fe-S cluster synthesis | 7 |  |  |  | X |
| 1703016 | - | 19 | MW_RS08410 | CsbD, σ^B^-controlled | 7 |  | X |  |  |
| 1950901 | - | 48 | MW_RS09625 | Glucoseamin-6-phosphate isomerase | 6 |  | X |  |  |
| 2206982 | - | 67 | MW_RS11175 | RNA polymerase, subunit delta | 6 | X |  |  |  |
| 2594589 | - | 115 | MW_RS13185 | Glucarate transporter | 6 |  |  |  | X |

*) Cluster extends into the upstream PSM β1 ORF.
